# Surgical management and functional outcomes of cranial infections requiring craniotomy or craniectomy: a retrospective cohort study

**DOI:** 10.3389/fsurg.2026.1850910

**Published:** 2026-06-22

**Authors:** Daniel W. Griepp, Shivum Desai, Heather Heitkotter, Rabia Ahmed, Avery Roe, Bryce K. Sarcar, Ishan Perera, Joshua Caskey, Jeffrey P. Turnbull, Julio Rosado Philippi, Elise J. Yoon, Ammar Alsalahi, James Dragonette, Anna T. K. Griepp, Clifford M. Houseman, Prashant Kelkar, Chad F. Claus, Boyd F. Richards, Daniel A. Carr

**Affiliations:** Department of Neurosurgery, Henry Ford Health Providence Hospital, Michigan State University College of Human Medicine, Southfield, MI, United States

**Keywords:** cranial infection, craniectomy, intraparenchymal abscess, outcomes, subdural empyema, surgical site infection

## Abstract

**Introduction:**

Cranial infections requiring surgical intervention—whether surgical site infections (SSI) after craniotomy/craniectomy or spontaneous infections such as subdural empyema and intraparenchymal abscess—pose significant management challenges. We present our 10-year institutional experience treating these cases at a high-volume neurosurgical center.

**Methods:**

Retrospective review of patients ≥18 years old who underwent craniotomy or craniectomy for non-catheter-related cranial infection between 2015 and 2025. Data on demographics, comorbidities, microbiology, surgical management, antibiotic use, revision surgeries, and functional outcomes (mRS at closest available follow-up to 12 months) were collected. Subgroup observations by infection etiology (SSI versus spontaneous) were examined.

**Results:**

Forty-three patients (27 men, 16 women; mean age 55.5 years) were included. Cultures were positive in 41/43 cases, most frequently isolating *Cutibacterium acnes, Staphylococcus aureus,* and *Streptococcus intermedius*. The median duration of intravenous antibiotics was 6 weeks. At median 11-month follow-up, 25 of 38 evaluable survivors (65.8%) achieved a good outcome (mRS 0–3). Poor outcomes (mRS 4–6 or ≥3 revision surgeries) occurred in 13 patients (34.2%) and were associated with higher Charlson Comorbidity Index (*p* < 0.02) and underlying high-grade glioma or metastatic disease (*p* = 0.04). Patients with SSI had a higher rate of craniectomy (76.2% vs. 18.2%, *p* < 0.001). Culture yield remained high regardless of whether broad-spectrum antibiotics were initiated before intraoperative sampling.

**Conclusion:**

This institutional series highlights that infectious involvement of a devascularized bone flap often necessitates craniectomy and increases the revision surgery burden. Functional outcomes were driven primarily by pre-infection comorbidities and underlying aggressive pathology rather than infection etiology alone.

## 1 Introduction

Cranial infections requiring surgical intervention continue to represent a formidable clinical problem, with reported incidence ranging from 0.5% to 19.8% (1–4). These infections include spontaneous processes such as subdural empyema and intraparenchymal abscess, which often arise from contiguous spread (e.g., sinusitis) or hematogenous dissemination (5–7), as well as surgical site infections (SSI) that develop after craniotomy or craniectomy performed for other intracranial pathology (8–10). Both entities frequently necessitate repeat craniotomy or craniectomy, prolonged courses of broad-spectrum antibiotics, and vigilant monitoring for recurrence (11, 12). Management can be particularly complex in patients with significant pre-existing comorbidities or ongoing oncologic treatment (2, 6, 13, 14).

Although the literature addressing each infection type separately is well established, real-world series that describe the full spectrum of surgically managed cranial infections within a single neurosurgical practice remain relatively limited (12, 15, 16). We therefore reviewed our consecutive 10-year experience treating these patients within a high-volume neurosurgical service. This report aims to share practical lessons regarding perioperative decision-making, bone-flap management, revision surgery burden, and long-term functional recovery in this challenging population.

## 2 Materials and methods

### 2.1 Study design and setting

This retrospective study was approved by the Henry Ford Health System Institutional Review Board (Protocol Number: 17716). Requirement for informed consent was waived due to the retrospective nature of the study. This manuscript was written following the Strengthening the Reporting of Observational Studies in Epidemiology (STROBE) guidelines (17). The primary objective was to describe our institutional experience with the surgical management of non-catheter-related cranial infections requiring craniotomy or craniectomy, including patient characteristics, microbiology, perioperative practices, and functional outcomes. As a secondary, exploratory assessment only, we examined potential differences according to infection etiology (surgical site infection versus spontaneous infection).

All patients, between May 2015 and May 2025, who underwent craniotomy or craniectomy for the indication of cranial SSI or spontaneous cranial infection, were identified for inclusion from retrospective chart review. Patients were eligible for the study if they were at least 18 years of age. Classification of infection was guided by the Centers for Disease Control and Prevention (CDC) guidelines for SSI events. Patients with a SSI were identified as having obvious skin or soft tissue infection, purulent wound discharge, or imaging findings concerning for infection in the presence of systemic symptoms. These patients were required to have their initial surgery performed at our institution and to have survived for at least 14 days after that surgery. Patients with spontaneous cranial infection (subdural empyema or intracranial abscess), requiring surgical intervention, were defined by having radiographic findings concerning infection, followed by obvious, frank pus encountered intraoperatively and/or growth of microbial species on wound culture. Superficial infection was primarily above or at the cortical gray matter, while deep infection was primarily subcortical. These characteristics were confirmed by reviewing the surgeon's notes, pre-operative imaging, and a detailed operative report. Exclusion criteria consisted of (1) isolated external ventricular drain infections; (2) shunt catheter related infections; (3) patients with isolated meningitis; (4) patients with infectious intracranial pathology, such as intracranial abscess, who were managed on intravenous (IV) antibiotics alone; (5) those who were deemed to be poor surgical candidates and thus not offered surgery based on futility.

### 2.2 Patient characteristics

Patient demographics, comorbidities using the Charlson Comorbidity Index (CCI), prior infections or hospitalizations, and imaging findings were collected through electronic medical record review. The “index surgery” was defined as the first procedure specifically performed for infection management, regardless of prior surgeries. For SSI cases, details of the initial (non-infectious) surgery—such as tumor resection, hematoma evacuation, or aneurysm clipping—were recorded, along with the number of days between the initial and index surgeries. Laboratory data, including erythrocyte sedimentation rate (ESR), white blood cell (WBC) count, and C-reactive protein (CRP), were collected when available. CRP greater than 10 mg/L was considered elevated (18). The index surgery was characterized by procedure (craniotomy vs. craniectomy), location (supratentorial vs. infratentorial and superficial vs. deep), and use of vancomycin powder. Microbiological data were extracted from wound, tissue, blood, and cerebrospinal fluid (CSF) cultures. Postoperative management variables included antibiotic type, duration, and administration (IV and/or oral), need for revision surgery, intensive care unit (ICU) and hospital length of stay, and delay of chemotherapy or radiation in the case of SSI after tumor resection. Functional outcomes were assessed using the modified Rankin Scale (mRS) at or closest to 12-month follow-up, with scores grouped as good (mRS 0–3) or poor (mRS 4–6). Patients were also considered to have a poor outcome if they required three or more revision surgeries due to persistent infection. This threshold was determined by consensus among our neurosurgical faculty, residents, and advanced practice providers. While patients with cranial pathology commonly require one or two additional procedures (e.g., delayed cranioplasty after trauma or repeat resection for recurrent meningioma), the need for three or more revisions is uncommon outside of refractory infection and is typically associated with protracted ICU stays, extended antibiotic therapy, and significant morbidity. Follow-up data were gathered via EMR review, clinic notes, and serial imaging (computed tomography or magnetic resonance imaging). All patients were contacted by phone at least twice, one week apart, to account for possible missing or inadequate follow-up information.

### 2.3 Surgical care

A standardized surgical protocol is used at our institution. All patients undergo scalp shaving (at least 2 centimeters beyond the planned incision margin) in the operating room. Skin preparation typically involves alcohol wash and dry followed by an iodine-based solution [DuraPrep (3M, St. Paul, MN, USA)]; however, in revision cases, such as in SSI, betadine scrub was substituted. Patients are draped in a sterile manner only after the solution has dried in either case. In elective cranial surgery, all patients receive a weight-appropriate, pre-operative dose of either cefazolin or vancomycin before incision. When infection is suspected, and cultures have not been speciated before surgery, this dose, in addition to empiric therapy, is traditionally held until intraoperative cultures may be obtained to facilitate accurate cultures. The decision to replace the bone flap is made intraoperatively, based on brain relaxation and presumed infectious involvement of the bone flap. If cranioplasty infection is suspected, the bone/implant is always removed. In routine cranial cases, a dural substitute is routinely used if the dura is unable to be primarily closed. Postoperative care is managed by a multidisciplinary team comprised of neurosurgery, infectious disease, neurocritical care, and internal medicine. Broad-spectrum IV antibiotics are initiated and tailored based on culture results. Discharge typically involves transition to home or rehabilitation with a peripherally inserted central catheter (PICC) for extended IV antibiotics. Follow-up is maintained through inpatient stay (if prolonged), clinic visits, rehabilitation reports, and research staff phone calls.

### 2.4 Statistical analysis

Univariate analyses were performed using Fisher's exact test and chi-square for nominal variables or the Mann–Whitney U test for nonparametric continuous variables, when appropriate. All analyses were performed using SPSS Version 29.0.2.0 (IBM Corp, Armonk, NY, USA). *P*-values less than 0.05 were considered statistically significant. All subgroup comparisons were considered hypothesis-generating.

## 3 Results

### 3.1 Patient characteristics and surgical management

During the study period, 43 consecutive patients (27 men and 16 women; mean age 55.5 years, range 18–81) met the inclusion criteria and were included in the analysis (Table 1). Overall cohort characteristics, presenting symptoms, and index surgical details are summarized in Table 1.

**Table 1:** Baseline characteristics.

Characteristic	Value
N	43
Age, mean ± SD (range)	55.5 ± 14.9 (18–81)
Male, *n* (%)	27 (62.8%)
Body mass index, mean ± SD (range)	28.5 ± 6.3 (19–42)
CCI, median (range)	2 (0–9)
Tobacco user, *n* (%)	10 (23.3%)
Diabetic, *n* (%)	7 (16.3%)
Primary initial intracranial pathology, *n* (%)	
Subdural empyema	6 (14.0%)
Intracranial abscess	16 (37.2%)
Tumor, meningioma	8 (18.6%)
Tumor, high grade glioma	4 (9.1%)
Tumor, other	5 (11.6%)
Vascular lesion	4 (9.1%)
Indication for index surgery, *n* (%)	
Subdural empyema	6 (14.0%)
Intracranial intraparenchymal abscess	10 (23.3%)
Unknown intracranial lesion	6 (14.0%)
SSI, deep infection	14 (32.6%)
SSI, superficial infection	7 (16.3%)
Clinical presenting signs of infection, *n* (%)	
Headache	14 (32.6%)
Altered mental status	11 (25.6%)
Fever	5 (11.6%)
Convulsions/seizure	12 (27.9%)
Other	1 (2.3%)
Pre-operative laboratory findings, *n* (%)	
WBC count (>10 G/L) (*n* = 24)	23 (95.6%)
CRP value (>10 mg/L) (*n* = 21)	11 (52.4%)
ESR value (>20 mm/hr) (*n* = 21)	9 (42.9%)

SSI, surgical site infection; SD, standard deviation; ESR, erythrocyte sedimentation rate; CRP, c-reactive protein; WBC, white blood cell; CCI, Charlson Comorbidity Index; index surgery is defined as the first surgery performed to treat infection.

The primary initial intracranial pathology was intracranial abscess in sixteen patients, meningioma in eight, subdural empyema in six, grade IV glioma in four, vascular lesion in four (intraparenchymal hemorrhage, subdural hemorrhage, arteriovenous malformation, trigeminal neuralgia), metastatic tumor in two, and non-meningioma, low-grade tumor in three. The primary symptoms at presentation included headache (14 patients), seizure (12), new motor weakness and/or facial droop (11), and altered mental status (11). Eight (18.6%) patients presented obtunded or in status epilepticus and required intubation at presentation to the emergency department (ED). Broad-spectrum IV antibiotics were administered immediately on arrival to the ED in 15 cases (35%) and held prior to obtaining intraoperative cultures in 28 cases (65%). Twenty-three patients (53.4%) underwent craniotomy while 20 (46.5%) underwent craniectomy as the index surgery. The approach was supratentorial in 39/43 (90.7%) cases, with nearly half affecting the left hemisphere (18/39, 47%). Infection was localized primarily deep in 27/43 (62.8%) cases. No incisions, in both initial and index surgeries, were hair-sparing. Vancomycin powder was used in 12/43 (28%) cases. External ventricular drain was left in place and eventually weaned in 7/43 (16.3%) cases.

Postoperatively, microbial culture resulted in all but two patients, one of whom had been started on broad-spectrum IV antibiotics before intraoperative culture. Thirty-four unique microbial species were cultured, 24 of which were only encountered in a polymicrobial infection (Table 2). Nineteen patients had polymicrobial infection (median 3 species, range 2–7). Antibiotic therapy was selected based on positive cranial wound cultures (*n* = 38), blood cultures (*n* = 2), CSF cultures (*n* = 1), and empirically (*n* = 2). The median duration of IV antibiotic treatment was 6 weeks. Recovery was complicated by either deep vein thrombosis (DVT) or pulmonary embolism (PE) in seven patients, and by sepsis or pneumonia in seven, two of which occurred concomitantly with DVT or PE. All but two patients required admission and management in the neuroscience ICU (mean 10.1 days ± 10.5, range 1–35) prior to management on the floor and ultimate discharge (days in hospital: mean 17.3 ± 10.5, range 4–96). Four patients (3 SSI and 1 spontaneous) in whom craniotomy was initially performed (5 SSI and 18 spontaneous) ultimately required return to OR for removal of the bone flap due to infectious involvement.

**Table 2:** Microbial species grown from cranial wound cultures.

Microbial species cultured	N	SSI, n	Spontaneous infection, n
Gram positive			
*Staphylococcus spp.*	11		
*Staphylococcus aureus*	7	5	2
*Staphylococcus epidermidis*	3	2	1
*Staphylococcus lugdunensis*^a^	1	1	0
*Streptococcus spp.*	11		
*Streptococcus intermedius*	6	0	6
*Streptococcus anginosus*^a^	3	0	3
*Streptococcus mitis oralis*^a^	1	1	0
*Streptococcus constellatus*^a^	1	0	1
*Cutibacterium acnes* ^a^	11	7	4
*Parvimonas micra* ^a^	5	0	5
*Actinomyces spp.*	3		
*Actinomyces meyeri*^a^	1	0	1
*Actinomyces israelii*^a^	1	0	1
*Actinomyces funkei*^a^	1	0	1
*Peptoniphilus harei* ^a^	2	0	2
*Arcanobacterium haemolyticum* ^a^	1	0	1
*Corynebacterium striatum* ^a^	1	0	1
Gram negative			
*Fusobacterium nucleatum*	7	0	7
*Klebsiella spp.*	4		
*Klebsiella pneumoniae*	2	2	0
*Klebsiella aerogenes*	1	1	0
*Klebsiella oxytoca*^a^	1	0	1
*Serratia marcescens*	3	3	0
*Pseudomonas aeruginosa*	2	1	1
*Aggregatibacter aphrophilus* ^a^	2	0	2
*Escherichia coli* ^a^	2	2	0
*Bacteroides fragilis* ^a^	1	0	1
*Bacteroides ovatus* ^a^	1	0	1
*Eikenella* ^a^	1	0	1
*Morganella morganii* ^a^	1	0	1
*Prevotella nigrescens* ^a^	1	0	1
*Prevotella bivia* ^a^	1	1	0
*Salmonella*	1	0	1
Fungal			
*Candida glabrata* ^a^	1	1	0
*Candida parapsilosis* ^a^	1	1	0
*Aspergillus fumigatus* ^a^	1	1	0
*Rhizopus*	1	0	1

spp, species.

a denotes microbial species only grown in polymicrobial infection.

Three patients (ages 57, 68, and 74 years) were transitioned to hospice and terminally weaned postoperatively (day 19, day 12, and day 14, 7% mortality). Among survivors, 4 patients required tracheostomy, although all were ultimately reversed. Fifteen patients were discharged to long-term acute care or rehab, while the remaining patients were discharged home. Twenty patients (46.5%) required at least one additional surgery after the index procedure for either cranioplasty or persistent infection, 10/43 (23.3%) patients required at least two, and 6/43 (14.0%) required three or more revision surgeries after the index procedure. Seventeen patients ultimately underwent cranioplasty: 15 were successful, while two required cranioplasty removal for persistent infection. One patient, with an infratentorial abscess, remained shunt dependent.

### 3.2 Infection etiology

Exploratory observations by infection etiology showed a significantly higher rate of craniectomy in SSI cases (76.2% vs. 18.2%, *p* < 0.001) and a nonsignificant trend toward more revision surgeries (Table 3). All other surgical characteristics, complications, length of stay, and antibiotic duration were comparable between the two groups. In the SSI group, the mean time from initial to index surgery was 40 days (standard deviation 27, range 10–119), while the initial surgery was the index surgery in the spontaneous group.

**Table 3:** Perioperative characteristics.

Characteristic	SSI	Spontaneous infection	p
N	21	22	
Female, *n* (%)	9 (42.8%)	7 (31.8%)	NS
Age, mean ± SD (range)	58 ± 11 (27–72)	53 ± 18 (18–81)	NS
CCI, median (range)	4 (0–9)	1 (0–7)	<0.001
Index surgery, *n* (%)			
Craniotomy	5 (23.8%)	18 (81.8%)	<0.001
Craniectomy	16 (76.2%)	4 (18.2%)	
Location of infection, *n* (%)			
Supratentorial surgery	18 (85.7%)	21 (95.5%)	NS
Deep infection	12 (57.1%)	17 (77.3%)	0.20
Dominant hemisphere	8 (38.1%)	10 (45.5%)	NS
Days from initial to index surgery (SSI only), mean ± SD (range)	40 ± 27 (10–119)	–	–
Presence of implant or foreign material, *n* (%)			
Bone flap	5 (23.8%)	18 (81.8%)	<0.001
External ventricular drain	1 (4.8%)	6 (27.3%)	0.09
Antibiotics held until OR cultures obtained, *n* (%)	17 (81%)	11 (50%)	0.06
Frontal sinus dehiscence, *n* (%)	0	4 (18.2%)	0.12
Vancomycin powder used, *n* (%)	7 (33.3%)	5 (22.7%)	NS
Chemotherapy or radiation delayed due to infection (SSI only), *n* (%)	6 of 6 (100%)	–	–
Postoperative Care			
Positive blood cultures, *n* (%)	2 (9.5%)	3 (14.3%)	NS
Positive CSF cultures, *n* (%)	2 (9.5%)	1 (4.5%)	NS
Polymicrobial infection	7 (33.3%)	12 (54.5%)	0.22
Length of ICU stay (days), mean ± SD	9.2 ± 12.9	10.7 ± 8.3	NS
Total length of stay (days), mean ± SD	17.0 ± 19.7	17.7 ± 11.1	NS
Duration of IV antibiotics in weeks, mean ± SD	5.55 ± 3.7	5.8 ± 2.5	NS
Surgical revision, *n* (%)			
Any revision surgery	13 (61.9%)	7 (31.8%)	0.07
Initial craniotomy required later revision to craniectomy	3 (14.3%)	1 (4.5%)	NS
Need for ≥3 revision surgeries after index surgery	5 (23.8%)	1 (4.5%)	0.10
Permanent CSF diversion required	0	1 (4.5%)	NS
Cranioplasty	13 of 16 (81%)	4 of 4 (100%)	NS
Cranioplasty attempted and removed for recurrence	2 of 13 (15%)	0	NS
Outcomes, *n* (%)			
Died while inpatient from infection	1 (4.3%)	2 of 22 (9.1%)	NS
Survivors who required tracheostomy	0 of 19	4 of 20 (20%)	NS
Discharged to rehab or long-term acute care	6 (28.6%)	9 (40.9%)	NS
Follow up (months), median (range)	11 (1–44)	6.5 (1–32)	–
Poor outcome (mRS 4–6 and/or ≥3 revision surgeries after index)	9 of 18 (50%)	4 of 20 (20%)	0.09

SSI, surgical site infection; SD, standard deviation; OR, operating room; ICU, intensive care unit; IV, intravenous; CSF, cerebral spinal fluid; mRS, Modified Rankin Scale; NS, not significant; CCI, Charlson Comorbidity Index; index surgery is defined as the first surgery performed to treat infection.

### 3.3 Outcomes

Of 43 patients, 3 died inpatient, leaving 40 survivors; 38 had sufficient outcome data for primary analysis. These patients were further grouped into good and poor outcomes, according to study criteria (Table 4). At last follow-up, 25 (9 SSI and 16 spontaneous) of 38 evaluable survivors (65.8%) achieved a good outcome. Poor outcome was associated in univariate analysis with higher CCI (*p* < 0.02) and underlying high-grade glioma or metastasis (*p* = 0.04). A higher (though nonsignificant) proportion of poor outcomes was noted in patients with SSI (50% vs. 20%, *p* = 0.09), consistent with the greater pre-infection disease burden in these cases.

**Table 4:** Predictive variables of good (mRS 0–3 and <3 surgeries after index) versus poor (mRS 4–6 and/or ≥3 revision surgeries after index) outcome.

Characteristic	Good	Poor	p
N	25	13	
Age, mean ± SD (range)	58.5 ± 14.4 (18–72)	58.9 ± 16.3 (18–66)	NS
CCI, median (range)	2 (0–6)	4 (0–9)	<0.02
SSI, high grade glioma or metastatic tumor pathology, *n* (%)	2 (8%)	5 (38.5%)	0.04
Spontaneous infection, *n* (%)	16 (64%)	4 (30.8%)	0.09
Deep infection, *n* (%)	17 (68%)	9 (69.2%)	NS
Vancomycin powder used, *n* (%)	5 (20.8%)	5 (46.2%)	NS
Polymicrobial infection, *n* (%)	10 (41.7%)	6 (46.2%)	NS

Analysis available for 38 patients (5 lost to follow-up).

SSI, surgical site infection; SD, standard deviation; mRS, Modified Rankin Scale; NS, not significant; CCI, Charlson Comorbidity Index.

## 4 Discussion

This 10-year institutional review describes our consecutive experience managing 43 patients who required craniotomy or craniectomy for cranial infection at a single high-volume neurosurgical service. The overall rate of good functional outcome (66% of evaluable survivors) and low infection-attributable inpatient mortality (7%) provide several practical lessons that may assist other centers facing similar cases.

The conversion rate from craniotomy to craniectomy was 17.4% overall (4 of 23), yet 60% in SSI cases. Notably, three of five SSI patients in whom craniotomy was initially performed ultimately required bone flap removal. This reinforces the importance of careful intraoperative assessment, as infectious involvement of a devascularized bone flap typically necessitates removal to eliminate any foreign body while the infection clears. As patients undergoing craniectomy will require future cranioplasty, this approach contributes to a higher rate of additional procedures and may influence long-term outcomes in SSI cases. In contrast, the absence of infectious bone involvement in spontaneous cases may contribute to a lower revision rate. Similar trends have been reported in prior case series on subdural empyema and brain abscess (6, 7, 19).

Poor outcomes were more frequent in patients with higher Charlson Comorbidity Index scores and underlying high-grade glioma or metastatic disease. Although interruption of oncologic therapy (chemotherapy or radiation) likely influenced outcomes, we could not quantify this effect. Among patients with spontaneous infection, all four poor outcomes occurred in cases where mass lesions were not initially recognized as infectious until surgery, suggesting that diagnostic delay may have played a role, although this could not be formally analyzed. Exploratory observations revealed no significant differences between groups in inpatient mortality, need for tracheostomy, or rehabilitation placement.

Regarding antibiotic timing, culture negativity occurred in only one case despite antibiotics being initiated prior to surgery in 15 patients (35%). This suggests that withholding broad-spectrum antibiotics until intraoperative sampling has minimal impact on culture yield in this population, provided the delay is not prolonged. We agree with the literature that prompt antibiotic administration is essential in patients presenting with sepsis or neurological compromise (20–23). The most frequently isolated organisms in this study (*Cutibacterium acnes*, *Staphylococcus aureus*, and *Streptococcus intermedius*) are consistent with previous reports (24–26). Our findings also highlight the ability of fungal pathogens to complicate cranial infections, which has been infrequently documented (8, 25).

This study has several limitations. Chief among them is its retrospective design, which renders it susceptible to selection and confounding biases. We were unable to include patients with SSI managed non-surgically, as they could not be reliably identified unless they later required surgery after failing conservative treatment. Furthermore, we recognize that comparing SSI and spontaneous cranial infections is to some degree inappropriate given their fundamentally distinct etiologies and the heavy confounding by underlying pathology (particularly aggressive malignancy in the SSI group). Thus, any observed differences should be interpreted cautiously as exploratory observations only. Additional limitations include inconsistent follow-up, which prevented standardized outcome assessment at fixed time points, and the relatively small sample size, which limited the ability to perform more robust statistical analyses. Any inferences drawn from the outcome data should therefore be interpreted with caution. Nevertheless, this cohort encompasses all surgically treated cases over a 10-year period and reflects the infrequency and clinical heterogeneity of cranial infections requiring operative intervention.

## 5 Conclusion

Taken together, this institutional experience emphasizes three clinically relevant points: infectious involvement of a devascularized bone flap in SSI cases frequently necessitates craniectomy and increases the likelihood of future revision surgeries; prompt empiric broad-spectrum antibiotics are appropriate and safe when patients present with signs of sepsis or neurological compromise, as withholding antibiotics until intraoperative sampling had minimal impact on culture yield; and functional outcomes are driven primarily by pre-infection comorbidity burden and underlying aggressive pathology rather than infection etiology alone.

## Data Availability

The original contributions presented in the study are included in the article/Supplementary Material, further inquiries can be directed to the corresponding author.
